# Successful Management of Spontaneous Unilateral Twin Ectopic Pregnancy With Two-Step Dose of Methotrexate

**DOI:** 10.1155/2024/5543780

**Published:** 2024-08-19

**Authors:** Lauren A. Forbes, Navya Nuthivana, Renee Morales

**Affiliations:** Department of Obstetrics and Gynecology Eastern Virginia Medical School at Old Dominion University, Norfolk, Virginia, USA

## Abstract

The incidence of unilateral tubal twin pregnancy is 1/20,000–1/250,000 with about 100 reported cases. Four of the six cases that were medically managed were successful. A 24-year-old female presented to the emergency department (ED) with vaginal bleeding and abdominal cramping. She was hemodynamically stable without signs of an acute abdomen. Laboratory evaluation revealed she was pregnant with a serum beta-human chorionic gonadotropin (b-hCG) of 798 mIU/mL. Transvaginal ultrasound (TVUS) revealed a single left tubal pregnancy with a yolk sac. The patient elected medical management with body surface area (BSA)–based intramuscular (IM) methotrexate (MTX). On Day 4, the patient returned to the ED; her b-hCG was 727 mIU/mL. TVUS revealed twin left tubal pregnancies with yolk sacs and fetal poles without cardiac activity. The patient elected to continue medical management with a second dose of BSA-based IM MTX. On Day 6, the patient returned to the ED with abdominal and rectal pain. She was hemodynamically stable without signs of an acute abdomen. Her b-hCG was 533 mIU/mL. TVUS showed persistent twin left tubal pregnancies—one at 5 weeks gestational age and the other at 6 weeks gestational age—without evidence of rupture. The patient continued serial b-hCGs. Thirty-one days after the first dose of MTX, her b-hCG was < 1 mIU/mL. TVUS showed resolution of tubal pregnancies. The patient consented to the publication of this case report. This case documents the successful treatment of spontaneous, unilateral tubal twin pregnancies with two-step dosing of IM MTX.

## 1. Introduction

An ectopic pregnancy is a pregnancy is implanted in an extrauterine location and may be a singleton or a multiple gestation. According to a recent study, the incidence of a unilateral twin tubal pregnancy is 1/20,000–1/250,000 [1]. At this time, one hundred and six cases of ectopic twin pregnancies have been reported. These cases are likely underreported as most of the cases are identified during surgical management. As such, options for management are also underreported and may be skewed towards surgical interventions.

Ectopic pregnancies can be managed expectantly, medically, or surgically. Expectant management is reserved for hemodynamically stable patients with a benign exam and decreasing or plateaued serum beta-human chorionic gonadotropin (b-hCG). Medical management as single-, double-, or multidose methotrexate (MTX) via intravenous, intragestation, or intramuscular (IM) administration is also reserved for stable patients. Coadministration of mifepristone [2, 3] or gefitinib [4] with MTX to improve treatment success rates has been studied. Surgical management is performed via salpingectomy or salpingostomy—typically via laparoscopy for stable patients or laparotomy for unstable patients. Emerging surgical techniques include laparoscopic Endoloop-assisted cornuectomy [5], uterine artery embolization [6], or hysteroscopic diode laser resection [7].

A study by Juneau and Bates showed no significant difference in success rates of expectant versus medical versus surgical management of singleton ectopic pregnancies among appropriately selected patients [8]. When medical management is pursued, there are mixed data on statistically significant differences in success rates between single-dose versus multidose MTX [8, 9]. Six of the one hundred and six cases of ectopic twin pregnancies were medically managed with varying route and dosing of MTX [1, 10–14]. Of those, four did not require further treatment with surgical management [10, 12–14].

## 2. Case Presentation

A 24-year-old female gravida 5 para 1214 presented to the emergency department (ED) with a single episode of vaginal bleeding associated with lower abdominal cramping. Her last menstrual period was 6 weeks and 2 days prior to presentation. She tested positive with a home pregnancy test 3 days prior to presentation. Her obstetrical history included three cesarean deliveries including the most recent 3 years prior for a dichorionic, diamniotic twin gestation. She reported an induced medical abortion 4 months prior to presentation. Her gynecological history included a history of chlamydia and an abnormal Pap smear greater than 5 years prior. She had no significant medical history, and her surgical history was only significant for the cesarean deliveries described above. The patient denied taking any medications. She reported 3 years of tobacco use quantified by five cigarettes a month as well as occasional marijuana use. On exam, the patient was afebrile with stable vital signs. The patient's abdomen was soft, nondistended, and without signs of rebound tenderness or guarding. She had mild tenderness to palpation at the epigastric area.

**Figure 1:**
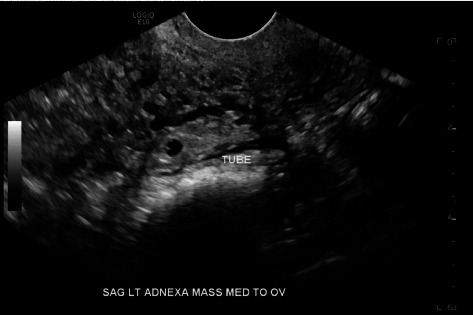
TVUS left adnexal ectopic pregnancy, sagittal view.

**Figure 2:**
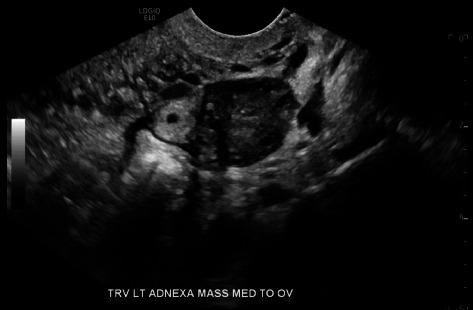
TVUS left adnexal ectopic pregnancy, transverse view.

In the ED, a urine pregnancy test was positive. Blood work showed serum b-hCG of 798 mIU/mL, hemoglobin of 12.2 g/dL, hematocrit of 35.8%, and an A positive blood type. Transabdominal ultrasound revealed a uterus measuring 10.23 cm in length and 3.36 by 5.27 cm in transverse dimensions (volume 179.20 mL) with no intrauterine gestational sac. Transvaginal ultrasound (TVUS) revealed an endometrium measuring 1.13 cm. A left adnexal ectopic pregnancy measuring 1.16 cm in length by 1.11 cm in width by 1.05 cm in height was identified in the fallopian tube, medial to the left ovary. The yolk sac measured 0.14 cm, and the gestation sac measured 0.40 cm, indicating a gestational age less than 5 weeks and 0 days. No fetal pole or fetal cardiac activity was noted (Figures 1 and 2). The right ovary was identified, and no right adnexal abnormalities were seen. There was minimal complex free fluid in the cul-de-sac.

The patient was counseled on medical versus surgical management of ectopic pregnancy. The patient elected for medical management and was counseled on absolute need for prolonged follow-up, risk of failure for medical management, and ruptured ectopic pregnancy precautions requiring the need for immediate medical evaluation. The patient received one dose of body surface area (BSA)–based IM MTX and instructed to return to the ED for repeat b-hCG on Days 4 and 7 following MTX administration or signs of rupture. The patient was also scheduled for a gynecology hospital follow-up appointment.

On Day 1, the patient presented to the ED with sporadic, crampy lower right quadrant abdominal pain associated with constipation. She reported scant vaginal bleeding requiring less than one pad since her initial presentation. On exam, the patient was afebrile with stable vital signs. The patient's abdomen was soft, nondistended, and without signs of guarding or rigidity. Mild tenderness to palpation was noted in the right lower quadrant as well as mild uterine tenderness.

Blood work showed a hemoglobin of 12.4 g/dL and hematocrit of 37.8%. A limited bedside transabdominal ultrasound performed by an ED physician revealed no intrauterine pregnancy or free fluid in the cul-de-sac. The patient was discharged home with a bowel regimen, reinstructed to return to the ED for signs of rupture and repeat b-hCG on Days 4 and 7 following MTX administration, and reminded of her gynecology hospital follow-up appointment.

**Figure 3:**
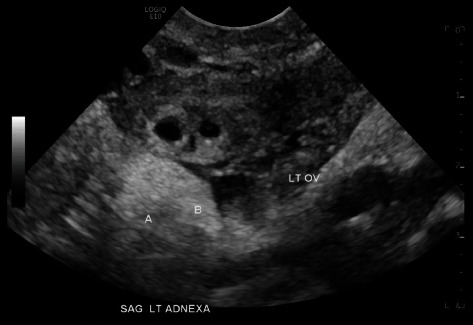
TVUS left adnexal ectopic twin pregnancy, sagittal view.

**Figure 4:**
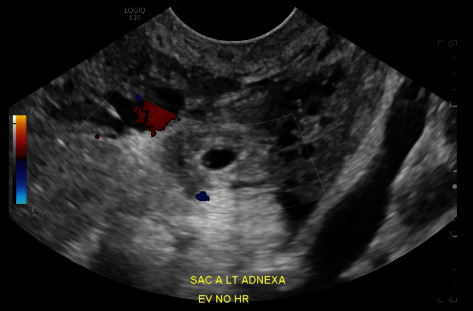
TVUS left adnexal ectopic twin pregnancy, sagittal view without cardiac activity.

**Figure 5:**
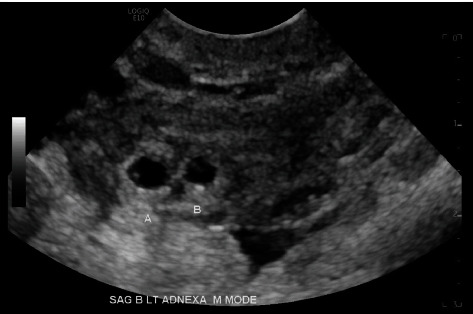
TVUS left adnexal ectopic twin pregnancy, sagittal view with M mode.

On Day 4, the patient presented to the ED for repeat b-hCG with mild vaginal bleeding. Blood work showed serum b-hCG of 727 mIU/mL, hemoglobin of 12.5 g/dL, and hematocrit of 38.7%. The ED physician ordered a repeat TVUS which revealed a persistent left adnexal ectopic pregnancy with interval development of a fetal pole and an adjacent gestational sac with a yolk sac and fetal pole (Figure 3). The radiologist identified the average gestational age as 5 weeks and 0 days; fetal cardiac activity was not noted (Figures 4 and 5). There was a small amount of free fluid in the cul-de-sac. The crown-rump length (CRL) in Sac A measured 0.15 cm, indicating a gestational age of 5 weeks and 0 days. A yolk sac was not identified. The yolk sac measured 0.1 cm, and the CRL measured 0.10 cm in Sac B indicating a gestational age of 5 weeks and 0 days. The ED physician consulted gynecology due to the interval findings. The patient was counseled on interval changes identified via imaging as well as the paucity of treatment or outcome data regarding her case. Further history revealed the patient's and her partner's family medical histories were significant for twin deliveries in addition to the patient's own twin gestation. The patient was again counseled on medical versus surgical management of ectopic pregnancy [15]. The patient strongly desired to avoid surgery and elected for continued medical management. She was recounseled on the absolute need for prolonged follow-up, theoretical increased risk of failure for medical management given significant interval development, and ruptured ectopic pregnancy precautions requiring the need for immediate surgery. The patient received a second dose of BSA-based IM MTX and was instructed to return to the ED for signs of rupture with repeat b-hCG on Day 7 following initial MTX administration.

On Day 6, the patient returned to the ED with abdominal and rectal pain. Blood work showed serum b-hCG of 533 mIU/mL. TVUS showed persistent twin left tubal pregnancies—one at 5 weeks gestational age and the other 6 weeks gestational age—without evidence of rupture. On exam, the patient was stable without signs of an acute abdomen. The patient was discharged with oral opioid medication for pain relief and instructed to return to the ED for signs of rupture.

The patient continued to obtain serial b-hCGs and was contacted at least weekly to review symptoms. Thirty-one days after the first dose of MTX, the patient's serum b-hCG was < 1 mIU/mL. TVUS showed resolution of tubal pregnancies. The patient consented to the publication of this case report.

## 3. Discussion

This case report documents successful management of a spontaneous, unilateral tubal twin pregnancy through two IM administrations of MTX with the second dose 4 days after the first. Medical management of tubal ectopic pregnancy includes evaluation for both absolute and relative contraindications to MTX, of which this patient had none [15]. The ultrasound showed absence of fetal heartbeats in both fetal sacs, the initial hCG was 798, and the pregnancy measured < 4 cm in size on TVUS. Given the patient's symptoms, we advised against expectant management. The patient repeatedly declined surgical management, citing a common fear of surgical management negatively impacting her future fertility [8, 16–18].

Our institution used BSA dosing of MTX. This dosing strategy follows Baradwan, Khan, and Al-Jaroudi, where successful management of twin tubal pregnancy was achieved through two IM MTX administrations of 50 mg/m^2^ with the second dose 7 days after the first [14]. Our dosing strategy differs from other reported cases of twin ectopic pregnancies by the number of doses required to achieve successful management as well as the route of administration. A case reported by Karadeniz, Dilbaz, and Özkan documented successful management after five serial administrations of IM MTX [12]. Finally, two cases reported unsuccessful management despite multiple administrations of IM MTX with one case requiring surgical management due to an acute abdomen and syncope [1, 11]. The increased cellular proliferation associated with twin versus singleton ectopic pregnancies aligns with the trend of requiring multidose MTX to achieve resolution [10].

In a case study on medical management of tubal ectopic pregnancies, successful medical management was obtained in 11 out of 12 cases with fetal cardiac activity by using a combination of intragestation and IM administration of MTX. The average starting b-hCG in these cases was 12,161 [19]. However, this series did not include any twin ectopic pregnancies. Fernandez et al. reported successful management of twin tubal pregnancy via ultrasound-guided intragestation MTX followed by IM administration 48 h later. Local administration of MTX may minimize the need for multidose IM administration by reducing the drug's volume of distribution but is riskier due to the significant vascularity of pregnancies [10]. Overall, there is still no consensus on the number of doses and route of administration of MTX for medical management of twin tubal pregnancy [9, 15, 20, 21]. Consensus on MTX administration may strengthen efforts to identify agents for coadministration [2, 3].

Although medical management of twin tubal pregnancies has been successful, surgical management via salpingectomy or salpingostomy is most common. The route of surgical management is often dictated by the patient's hemodynamic stability, prior surgical history, and/or surgeon preference. Although alternate surgical techniques have successfully treated singleton ectopic pregnancies [5–7], their effectiveness has not been studied in twin ectopic pregnancies.

Whether salpingectomy or salpingostomy is superior regarding future fertility and whether surgical management is superior to medical management is heavily debated [8, 9, 15, 16, 22]. This patient strongly preferred medical management to retain her fallopian tubes as a form of fertility preservation. Nonetheless, residual fallopian tube adhesions may lead to ectopic pregnancy recurrence, and the patient may benefit from hysterosalpingography to assess tubal patency.

## 4. Conclusions

This case documents the success of two doses of IM MTX for medical management of spontaneous, unilateral tubal twin pregnancies.

## Data Availability

No underlying data was collected or produced in this study.
